# Supplementary material for: Conceptualizing the COVID-19 Pandemic: Perspectives of Pregnant and Lactating Women, Male Community Members, and Health Workers in Kenya
Source: Int J Environ Res Public Health. 2022 Aug 30;19(17):10784. doi: 10.3390/ijerph191710784 (PMC9518350; doi:10.3390/ijerph191710784)
Supplement: Supplementary file 1 [file ijerph-19-10784-s001.zip › ijerph-1843326-supplementary/SuppMaterial_InterviewGuide_Community.pdf]

IRB Office Use Only	IRB Approval Date:<br>IRB Consent Version No.:

JOHNS HOPKINS BLOOMBERG SCHOOL OF PUBLIC HEALTH

DATA COLLECTION FORM FOR KEY INFORMANT INTERVIEWS:
Semi-Structured Interview Guide, Community Members

Study Title: COVID 19: Preparing Early Adopter Countries for Maternal Vaccination
Principal Investigator: Limaye
IRB No.: IRB00014893
PI Version Date: February 22, 2021 – Version 1

INSTRUCTIONS TO FACILITATOR:
We are very interested in understanding perceptions related to COVID-19 vaccination of pregnant women and women who are currently lactating. The questions below serve as a guide for this discussion. The aim of this interview is to obtain attitudes related to COVID-19 vaccines and pregnancy. Therefore, we would like to get insights from different stakeholders related to COVID vaccination of pregnant women and women who are currently lactating, including pregnant women, their partners, family members, community members, and others.

GENERAL QUESTIONS:

1) I'd like to hear a little bit about you.

   Probes:
   - *Where are you from, and where do you live now?*
   - *Are you around 18-25? 25-45? 45-60? Older than 60? [capture age range]*
   - *Are you married? If so, how long have you been married?*
   - *[If pregnant or partner of pregnant woman] Is this your first pregnancy?*
   - *[If not first pregnancy] How many children do you have?*

2) Tell me about your household.

   Probes:
   - *How many people live in your home?*
   - *How are they related to you?*
   - *Do you have any family members who are pregnant?*
   - *Tell me about what you do during a typical day.*

3) Have you ever been ill with COVID-19 or has someone you know ever been ill with this disease?

   Probes:
   - *What have you heard about COVID? [insert local term/language]*
   - *What symptoms did you/they experience?*
   - *How long did you/they feel ill?*
   - *How sick did you/they feel? How severe was your/their illness?*
   - *How did your/their illness impact your/their daily activities and responsibilities?*
   - *[if never ill]: Do you view COVID-19 as a disease that you may come into contact with during your lifetime or not? Why?*
   - *How does your community view people who have been ill with COVID?*

4) Do you think that COVID is a problem in your community?

Probes:

- *Why or why not?*
- *Do you know of any efforts to control COVID in your community? If so, what are they?*
- *Do you know what you can do to protect yourself from COVID?*
- *Do you know where you could go if you need help with preventing COVID disease?*
- *Do you think pregnant and lactating women are more at risk of developing serious disease from COVID?*
    - a. *Why or why not?*

5) Tell me about what your community and/or the government has done to slow the spread of COVID.

Probes:

- *Do you think your community/government has done all it can to protect pregnant women?*
    - *Why or why not?*
- *Do you think your community/government has done all it can to protect health workers?*
    - *Why or why not?*
- *Do you think your community/government has done all it can to protect older community members?*
    - *Why or why not?*
- *How could the community/government improve their efforts?*
- *Are there other groups you think the community/government should focus on for protection against COVID-19? If so, can you tell me which groups and why?*

This next set of questions is about vaccines. I'd like to know more about your experience with vaccines.

6) Have you ever been vaccinated?

Probes:

- *At what point in your life? Why [if relevant]?*
- *[If pregnant or had children] Have you ever gotten vaccinated during pregnancy?*
    - *Do you remember which vaccines you received?*
    - *Tell me about your experience getting vaccinated during pregnancy*
- *Some vaccines, like tetanus vaccines, are recommended for pregnant women to help protect both the mother and the baby. What is your opinion on pregnant women getting vaccinated?*
- *If you have heard about vaccination during pregnancy, how did you hear? From whom?*
    - *Do you have any questions about vaccinating pregnant women?*
- *What are the biggest obstacles and challenges learning about and accessing vaccines?*
    - *Access to healthcare, distance and time to clinic, cost of transport, supply/availability, cost/willingness to pay*

7) Who do you go to for health information?

Probes:

- *If you were making a decision about vaccines, who would you go to for information?*
- *What about for COVID vaccines? Who would you go to for information?*
    - *Religious leaders?*
    - *Family members? Partner? Mother-in-law?*

- *Government officials or other community leaders?*
- *Health workers or [term for CHW]?*

- *Do you get information about health and vaccination from media or social media?*
  - *If so, tell me about which sources you rely on.*

8) Have you heard about the COVID vaccine?

Probes:

- *Where did you learn about the COVID vaccine?*
  - *Did you hear about it from your doctor? The media? A family member? Somewhere else?*
- *Do you know where you could get the COVID vaccine?*
- *Do you plan to get that COVID vaccine for yourself? For your family members? Tell me why or why not.*

9) Tell me more about your decision to be vaccinated. What are you thinking about in deciding whether or not to get the COVID vaccine?

Probes:

- *If you had COVID-19 or know someone who did, are you more or less likely to get the COVID-19 vaccine?*
- *If you were able to be vaccinated now, what information do you need to make an informed decision?*
  - *Who would you talk to?*
  - *What questions would you ask?*
- *Do you know if your family members plan to be vaccinated? Members of your community?*
  - *Tell me a little bit about how the community feels about COVID-19 vaccines.*
- *What do you know about vaccinating pregnant women with the COVID vaccine?*
- *Do you have any questions about vaccinating pregnant women with the COVID vaccine?*

[Ask only if the vaccine has been introduced in the country/province/county]

10) Has your community started to give the COVID-19 vaccine?

Probes (if yes):

- *What groups are currently being vaccinated in your community?*
  - *Do you know when you will be able to get the vaccine?*
- *Do you know anyone who has been vaccinated already?*
  - *Do you know any pregnant women who have been vaccinated?*
  - *[For pregnant women] Has your partner been vaccinated? If not, do they plan to?*
- *[If pregnant] Were you told you could not be vaccinated?*
  - *Do you know why they were told they could not be vaccinated?*
- *[If pregnant partner or family member] Was your pregnant partner/ family member told they could not be vaccinated?*
  - *Do you know why they were told they could not be vaccinated?*
