# Supplementary material for: Conceptualizing the COVID-19 Pandemic: Perspectives of Pregnant and Lactating Women, Male Community Members, and Health Workers in Kenya
Source: Int J Environ Res Public Health. 2022 Aug 30;19(17):10784. doi: 10.3390/ijerph191710784 (PMC9518350; doi:10.3390/ijerph191710784)
Supplement: Supplementary file 1 [file ijerph-19-10784-s001.zip › ijerph-1843326-supplementary/SuppMaterial_InterviewGuide_Providers.pdf]

IRB Office Use Only	IRB Approval Date:<br>IRB Consent Version No.:

JOHNS HOPKINS BLOOMBERG SCHOOL OF PUBLIC HEALTH

DATA COLLECTION FORM FOR KEY INFORMANT INTERVIEWS:
Semi-Structured Interview Guide, Healthcare Providers

Study Title: COVID 19: Preparing Early Adopter Countries for Maternal Vaccination
Principal Investigator: Limaye
IRB No.: IRB00014893
PI Version Date: February 22, 2021 – Version 1

Please note that no vaccines currently exist for RSV or group B strep. Therefore, questions regarding the "RSV vaccine" or the "group B strep" vaccine are theoretical, in order to gauge potential opinions and behaviors towards these vaccines which are currently in development. Participants are encouraged to refuse answering such questions if they do not feel comfortable addressing questions regarding potential future RSV and/or group B strep vaccines.

1) I'd like to hear a bit about you.
   Probes:
   - *What is your professional position? Can you tell me a bit about your work experience?*
   - *How long have you been working in the field?*
   - *What is your experience in maternal immunization or maternal health? How many pregnant patients do you regularly see?*

2) Have you ever been ill with COVID-19 or has someone you know ever been ill with this disease?
   Probes:
   - *What have you heard about COVID? [insert local term/language]*
   - *What symptoms did you/they experience?*
   - *How long did you/they feel ill?*
   - *How sick did you/they feel? How severe was your/their illness?*
   - *How did your/their illness impact your/their daily activities and responsibilities?*
   - *[if never ill]: Do you view COVID-19 as a disease that you may come into contact with during your lifetime or not? Why?*
   - *How does your community view people who have been ill with COVID?*

3) Do you think that COVID is a problem in your community?
   Probes:
   - *Why or why not?*
   - *Have you seen/treated many COVID-19 patients?*
   - *Do you know of any efforts to control COVID in your community? If so, what are they?*
   - *Do you know what you can do to protect yourself from COVID? Your patients?*
   - *Do you know where you could go if you need help with preventing COVID disease?*

- *Do you think pregnant and lactating women are more at risk of developing serious disease from COVID?*
    - *Why or why not?*

4) Tell me about what your community and/or the government has done to slow the spread of COVID.
   Probes:
   - *What positive actions has your community/government taken to protect pregnant women, is there more that could have been done?*
       - *Why or why not?*
   - *What positive actions has your community/government taken to protect health worker, is there more that could have been done?*
       - *Why or why not?*
   - *What positive actions has your community/government taken to protect older community members, is there more that could have been done?*
       - *Why or why not?*
   - *How could the community/government improve their efforts?*

This next set of questions is about vaccines. I'd like to know more about your experience with vaccines.

5) Have you ever been vaccinated?
   Probes:
   - *At what point in your life? Why [if relevant]?*
   - *What information sources influence your knowledge and opinions on vaccines?*
   - *[If pregnant or had children] Have you ever gotten vaccinated during pregnancy?*
       - *Do you remember which vaccines you received?*
       - *Tell me about your experience getting vaccinated during pregnancy*
   - *Some vaccines, like tetanus vaccines, are recommended for pregnant women to help protect both the mother and the baby. What is your opinion on pregnant women getting vaccinated?*

I want to ask you a few questions about how you view vaccines as a health worker. *Probes:*
- *As you are a health worker, how does your role as one affect your opinion of vaccines, if at all? Meaning, how do you feel about vaccines?*
- *Do you recommend vaccines generally to your patients?*
- *Does your workplace encourage vaccination?*
    - *Among staff? Among patients?*

6) Do you recommend vaccination for your pregnant patients?
   Probes:
   - *What vaccines do you recommend for pregnant patients?*
       i. Tetanus?
   - *Where do your pregnant patients gather information on vaccines?*
   - *Where do your pregnant patients receive vaccines?*
   - *Do your pregnant patients arrive with questions and/or concerns about vaccines?*

   i. *How do you typically handle it when a pregnant patient has concerns about vaccines?*
- *What are the biggest challenges for your patients in accessing vaccines?*
   i. *Access to healthcare, distance and time to clinic, cost of transport, supply/availability, cost/willingness to pay*

7) Who do you go to for health information?
   Probes:
   - *If you were making a decision about vaccines, who would you go to for information?*
   - *What about for COVID vaccines? Who would you go to for information?*
     - *Religious leaders?*
     - *Family members? Partner? Mother-in-law?*
     - *Government officials or other community leaders?*
     - *Health workers or [term for CHW]?*
   - *Do you get information about health and vaccination from media or social media?*
     - *If so, tell me about which sources you rely on.*
- *For vaccination of pregnant women, are there other information sources different than those mentioned, that you use?*

8) Have you heard about the COVID vaccine?
   Probes:
- *Where did you learn about the COVID vaccine?*
   i. *Peers? Professional community? News? Social media? A family member? Somewhere else?*
   ii. *Do you think this is also where your patients learn about the COVID vaccine?*
- *Do you know where you and/or your patients could get the COVID vaccine?*
- *Do you plan to get the COVID vaccine for yourself? For your family members? Tell me why or why not.*
- *Tell me a bit about whether or not you will recommend COVID-19 vaccination to your patients? What about to your pregnant patients? Would you recommend it? Why or why not?*

9) Tell me more about your decision to be vaccinated. What are you thinking about in deciding whether or not to get the COVID vaccine?
   Probes:
   - *If you had COVID-19 or know someone who did, are you more or less likely to get the COVID-19 vaccine?*
   - *If you were able to be vaccinated now, what information do you need to make an informed decision?*
     - *Who would you talk to?*
     - *What questions would you ask?*
   - *Do you know if your family members plan to be vaccinated? Members of your community?*
     - *Tell me a little bit about how the community feels about COVID-19 vaccines.*
   - *Do you have any questions about vaccinating pregnant women with the COVID vaccine?*
   - *Which information sources do you see as most important, influential or trustworthy to help decide whether pregnant women should receive the COVID vaccine?*

[Ask only if the vaccine has been introduced in the country/province/county]

10) Has your community started to give the COVID-19 vaccine?

Probes (if yes):

- *What groups are currently being vaccinated in your community?*
    - *Do you know when you will be able to get the vaccine?*
- *Do you know anyone who has been vaccinated already?*
    - *Do you know any pregnant women who have been vaccinated? Can you tell me about their experience?*

11) Are pregnant and lactating women (including health care workers) screened out and/or turned away during COVID-19 vaccination distribution campaigns in your community/country? Tell me a little about what you have seen or heard.

Probes:

- *How is COVID-19 vaccination information being communicated to pregnant and lactating women?*
- *Have pregnant or lactating women arrived at distribution sites expecting to be eligible to receive a COVID-19 vaccine? How do they react when they realize they are ineligible?*
- *Is there any distinction between pregnant women who are in a high-risk occupation or who have a chronic illness, compared to other pregnant women?*
